# Supplementary material for: Does co-expression of Yarrowia lipolytica genes encoding Yas1p, Yas2p and Yas3p make a potential alkane-responsive biosensor in Saccharomyces cerevisiae?
Source: PLoS One. 2020 Dec 17;15(12):e0239882. doi: 10.1371/journal.pone.0239882 (PMC7745969; doi:10.1371/journal.pone.0239882)
Supplement: S1 Fig — (DOCX) [file pone.0239882.s001.docx]

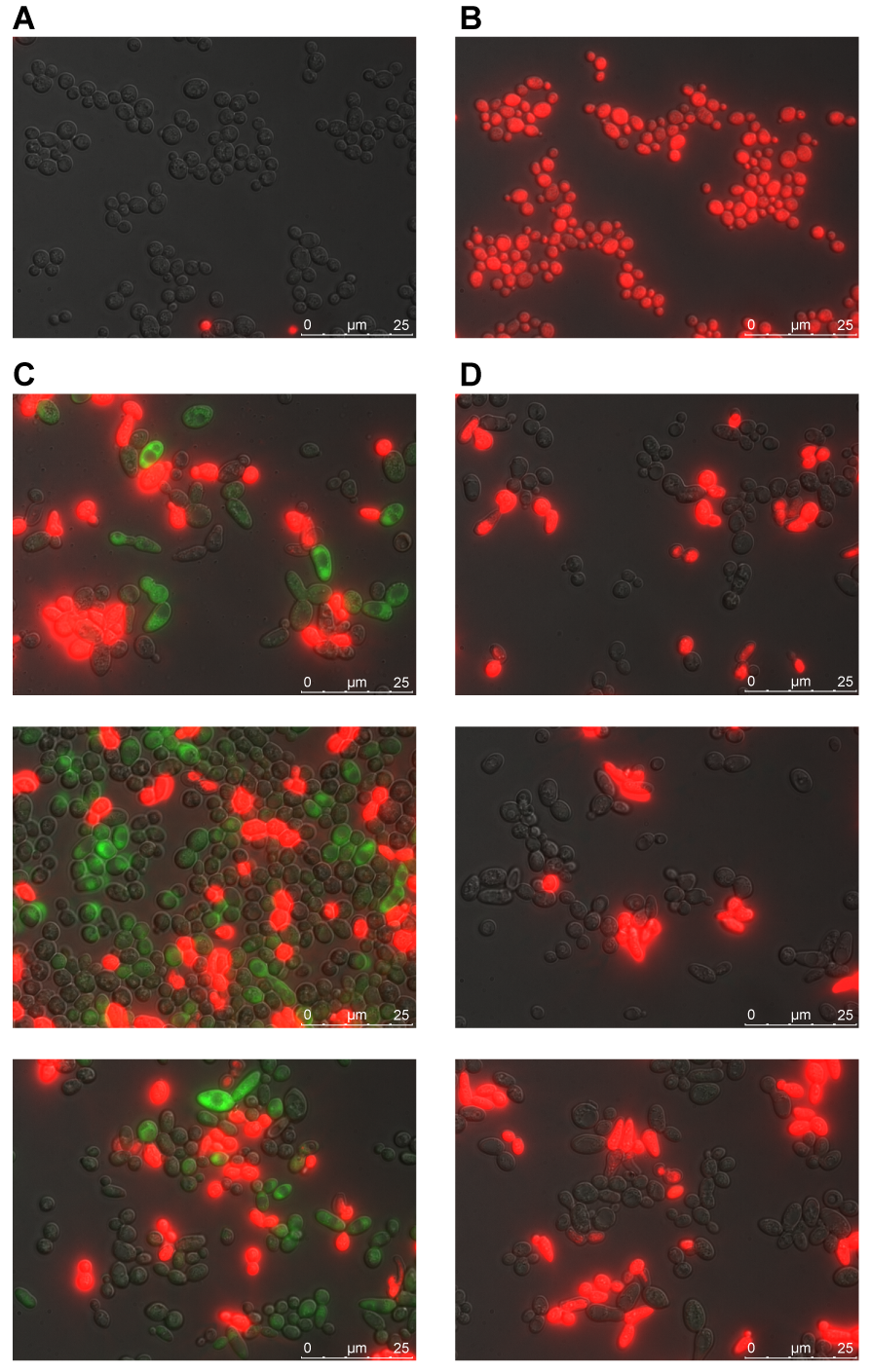


**S1 Fig. Propidium iodide (PI) staining.** A) Empty plasmid (live cells), B) Empty plasmid (dead cells, PI staining), C) P_ALK1_-GFP_Yas1p_Yas2p_Yas3p, D) P_CYC1__Yas1p_Yas2p_Yas3p. The strains were cultivated for 28 h after inoculation at OD 0.1 in shake flasks using synthetic complete media without uracil and histidine. Biological triplicates were evaluated employing fluorescence microscope. In C and D, overlay-pictures are seen with both GFP and RFP filter.
