# Supplementary material for: Does co-expression of Yarrowia lipolytica genes encoding Yas1p, Yas2p and Yas3p make a potential alkane-responsive biosensor in Saccharomyces cerevisiae?
Source: PLoS One. 2020 Dec 17;15(12):e0239882. doi: 10.1371/journal.pone.0239882 (PMC7745969; doi:10.1371/journal.pone.0239882)

**S2 Fig. Evaluation of Yas1p, Yas2p and Yas3p with the *Yarrowia lipolytica* promoter *ALK1*.** The transcription factors Yas1p, Yas2p and Yas3p derived from *Yarrowia lipolytica* were expressed in *S. cerevisiae* using endogenous promoters *PGK1* for expressing *YAS1* and *YAS2* and promoter *TEF1* for expressing *YAS3*. Promoter *ALK1* was placed upstream of GFP and interaction of the TFs with P*_ALK1_* was evaluated by measuring the fluorescence signal employing flow cytometry. Strains were cultured in synthetic complete media in shake flasks and measured for fluorescence and OD 6 h after inoculation. n = 3, error bar = ± SD.

**p*-value <0.05, ***p*-value <0.01 (Student’s *t* test)


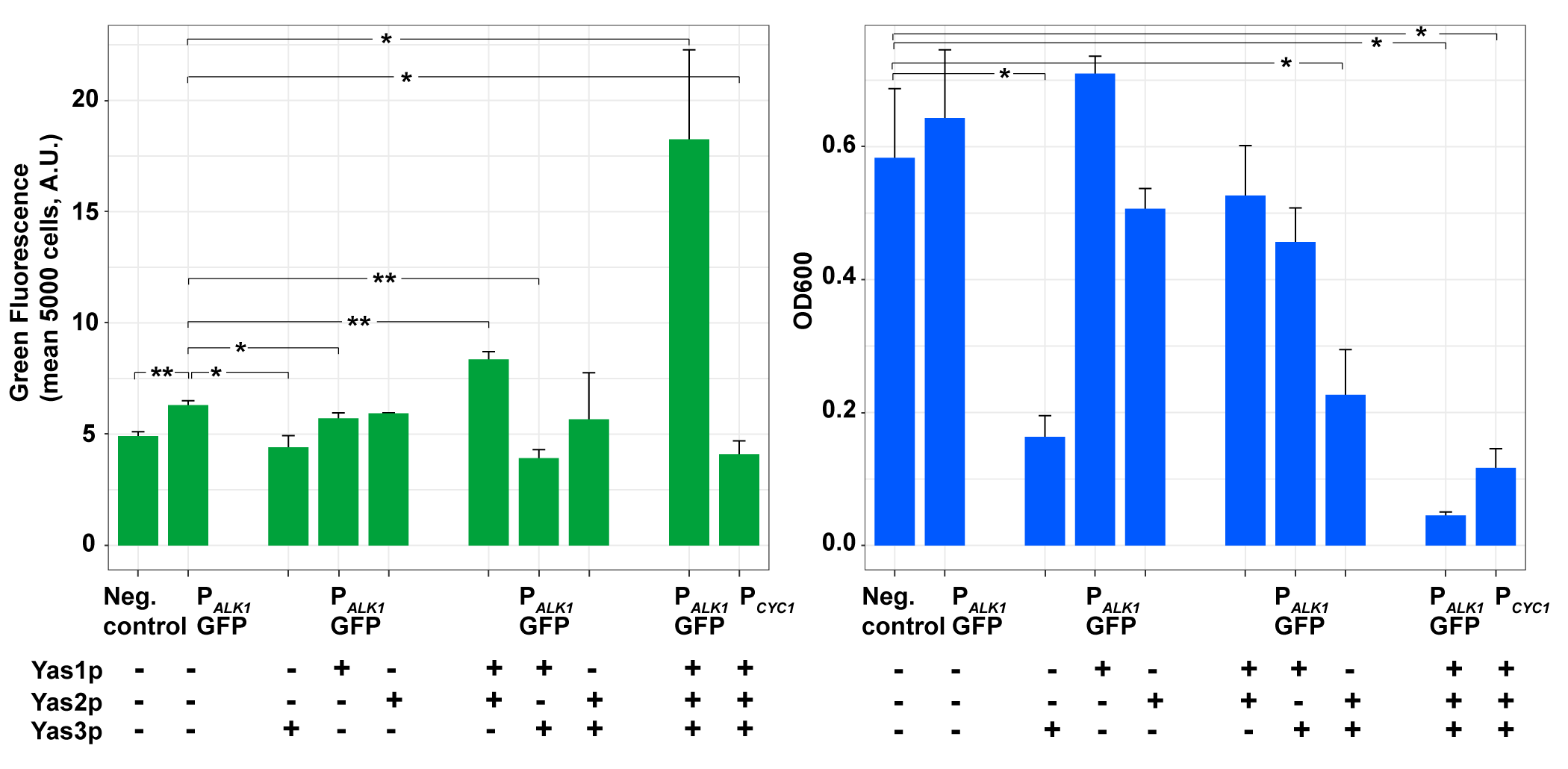

Supplement: S2 Fig — (DOCX) [file pone.0239882.s002.docx]
