# Supplementary material for: Does co-expression of Yarrowia lipolytica genes encoding Yas1p, Yas2p and Yas3p make a potential alkane-responsive biosensor in Saccharomyces cerevisiae?
Source: PLoS One. 2020 Dec 17;15(12):e0239882. doi: 10.1371/journal.pone.0239882 (PMC7745969; doi:10.1371/journal.pone.0239882)

**S3 Fig. Evaluation of Yas1p and Yas2p together with the endogenous promoter CYC1 as well as a synthetically modified version with ARE1 binding sites.** Genes encoding Yas1p and Yas2p were expressed in S. cerevisiae using the promoter CYC1 upstream of GFP. Strains were cultured in synthetic complete media in shake flasks and measured for fluorescence employing flow cytometry and OD 6 h after inoculation. Schematic of CYC1 promoters (orange/blue) containing the ARE1 BS(s) are shown under each pair of bar graph. n = 3, error bar = ± SD.

**p*-value <0.05, ***p*-value <0.01 (Student’s *t* test)


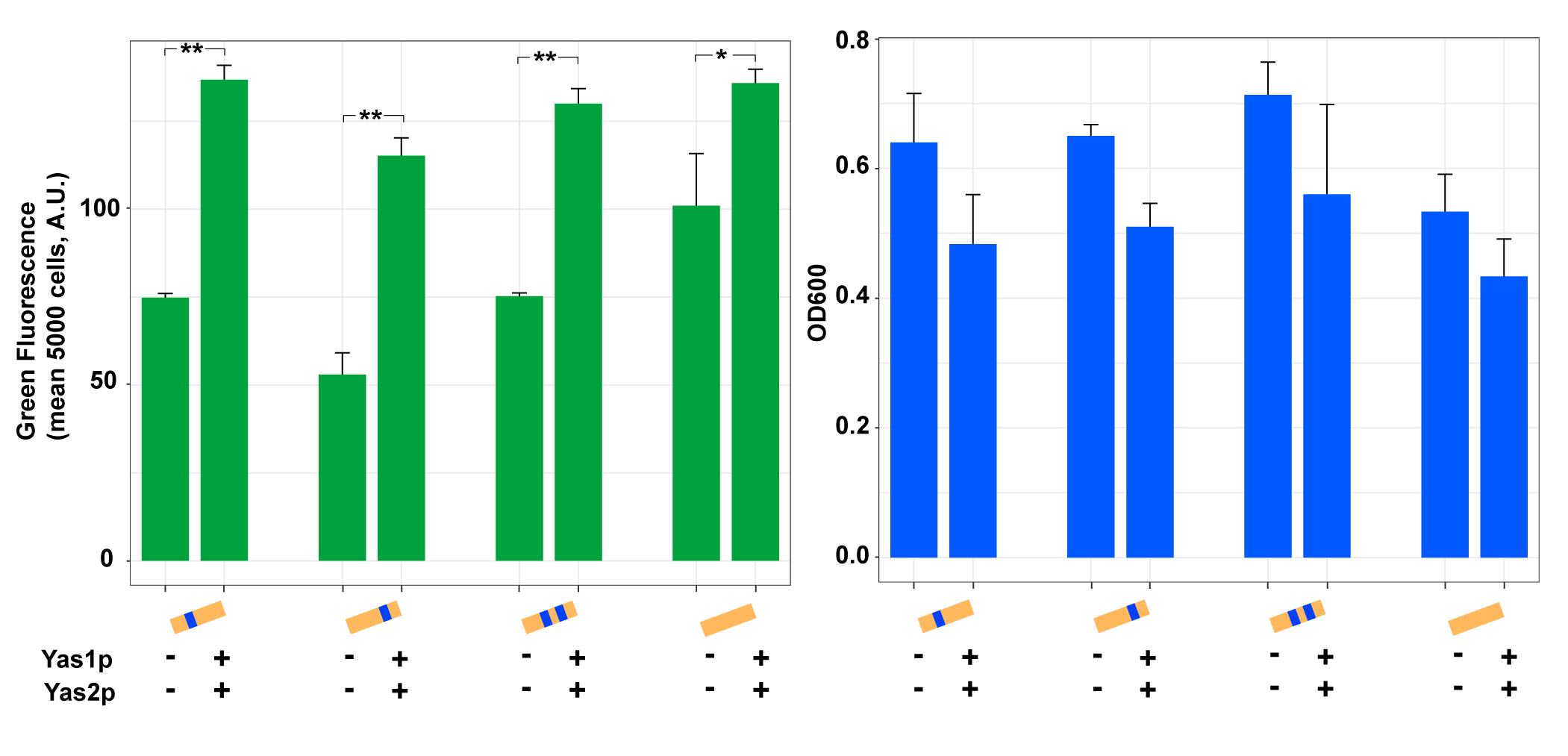

Supplement: S3 Fig — (DOCX) [file pone.0239882.s003.docx]
