# Supplementary material for: Does co-expression of Yarrowia lipolytica genes encoding Yas1p, Yas2p and Yas3p make a potential alkane-responsive biosensor in Saccharomyces cerevisiae?
Source: PLoS One. 2020 Dec 17;15(12):e0239882. doi: 10.1371/journal.pone.0239882 (PMC7745969; doi:10.1371/journal.pone.0239882)

**S4 Fig. Fusion of TFs Yas1p, Yas2p and Yas3p with GFP.** The TFs Yas1p, Yas2p and Yas3p were fused with GFP to evaluate their expression and localization. The fusion proteins were formed through a linker consisting of amino acids GGGS. A) The TFs were fused through their C-terminus to GFP. B) Yas1p fused to GFP under the control of P_PGK1_. C) Yas2p fused to GFP under the control of P_PGK1_. D) Yas3p fused to GFP under the control of P_TEF1_. E) An NLS tag was linked to the TFs, which were fused through their C-terminus to GFP. F) Yas1p with an NLS tag fused to GFP under the control of P_PGK1_. G) Yas3p with an NLS tag fused to GFP under the control of P_TEF1_. Samples were evaluated 6 h after inoculation.


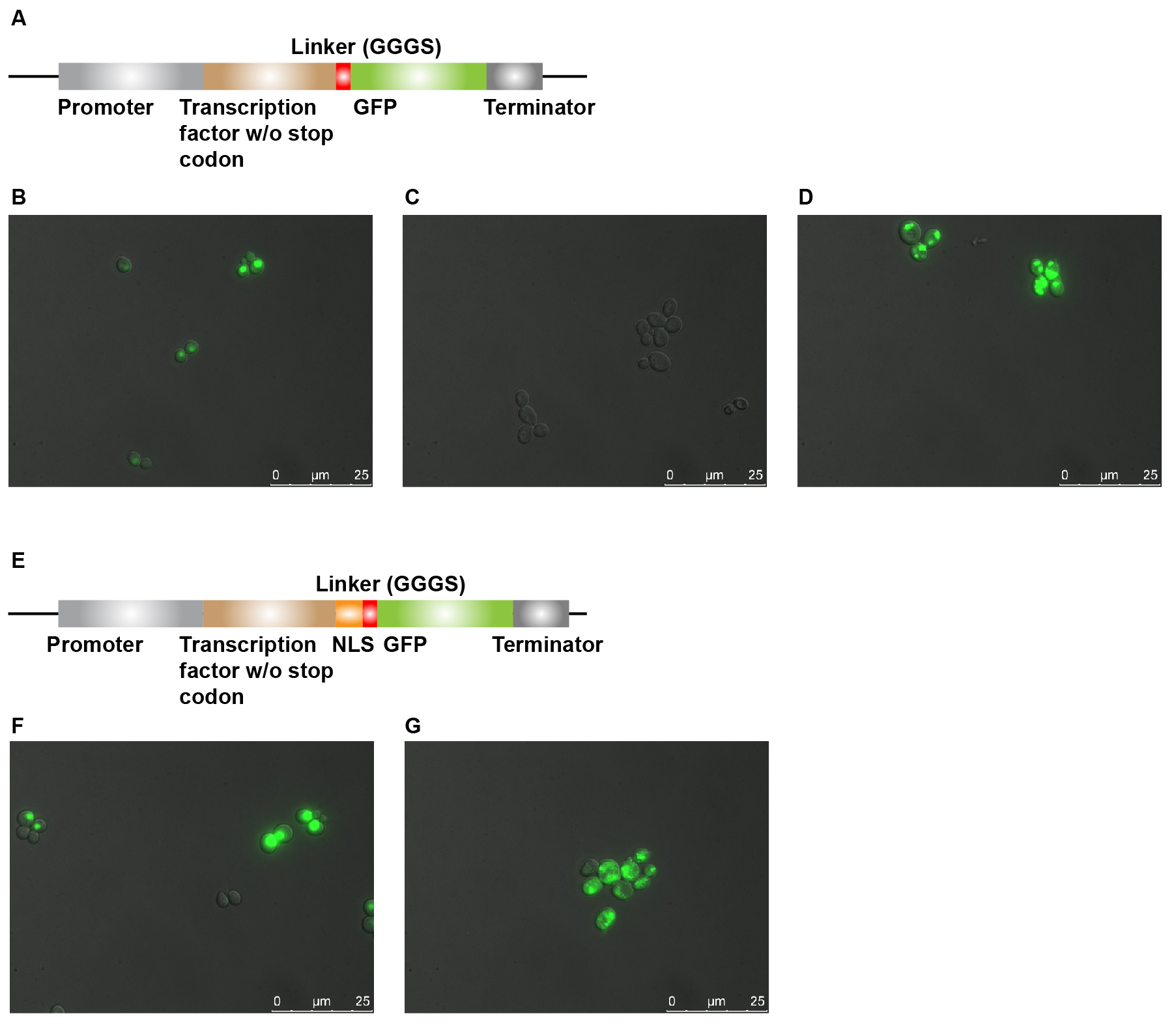

Supplement: S4 Fig — (DOCX) [file pone.0239882.s004.docx]
