# Supplementary material for: Does co-expression of Yarrowia lipolytica genes encoding Yas1p, Yas2p and Yas3p make a potential alkane-responsive biosensor in Saccharomyces cerevisiae?
Source: PLoS One. 2020 Dec 17;15(12):e0239882. doi: 10.1371/journal.pone.0239882 (PMC7745969; doi:10.1371/journal.pone.0239882)
Supplement: S1 Table — (DOCX) [file pone.0239882.s005.docx]

**S1 Table. Primers used in this study**

| **Name** | **Description** | **Sequence^1^** |
| --- | --- | --- |
| pYDA01 | p413TEF1_F | TCATGTAATTAGTTATGTCACGC |
| pYDA02 | p413TEF1_R | TTTGTAATTAAAACTTAGATTAGATTGCTATG |
| pYDA03 | GFP_F1 | tagcaatctaatctaagttttaattacaaaATGCGAATCCCCGGGTTAATTAAC |
| pYDA04 | GFP_R1 | aatgtaagcgtgacataactaattacatgaCTATTTGTATAGTTCATCCATGCCATG |
| pYDA05 | p413TEF1_R2 | GAGCTCCAGCTTTTGTTC |
| pYDA06 | P*_CYC1_*_F | cctcactaaagggaacaaaagctggagctcAATTTTTTTGGAAAACCAAG |
| pYDA07 | P*_CYC1_*_R | aatgtaagcgtgacataactaattacatgaTATTAATTTAGTGTGTGTATTTGTGTTTG |
| pYDA08 | GFP_F2 | ATGCGAATCCCCGGGTTAATTAAC |
| pYDA09 | P*_CYC1_*_R2 | tttactgttaattaacccggggattcgcatTATTAATTTAGTGTGTGTATTTGTGTTTG |
| pYDA10 | P*_ALK1_*_F | ctcactaaagggaacaaaagctggagctcCAGTGATGAGGACACACTC |
| pYDA11 | P*_ALK1_*_R | tactgttaattaacccggggattcgcatAGTGCAGGAGTATTCTGGG |
| pYDA12 | P*_CYC1_*_F2 | ccgccaggcgtgtatatatagcgtggatggCCAGGCAACTTTAGTGCTG |
| pYDA13 | P*_CYC1_*_R3 | CCATCCACGCTATATATACAC |
| pYDA14 | P*_PGK1_*_*YAS1*_t*_ADH1_*_F | tatacacatgtatatatatcgtatgctgcaACGCACAGATATTATAACATCTGC |
| pYDA15 | P*_PGK1_*_*YAS1*_t*_ADH1_*_R | agactgttgttcagccacgtcaaCATGCCGGTAGAGGTGTG |
| pYDA16 | *YAS2*_t_PDC6__F | attgaccacacctctaccggcatgTTGACGTGGCTGAACAAC |
| pYDA17 | *YAS2*_t_PDC6__R | ctactttttacaacaaatataacaaaATGCACTTATCCCATCCTC |
| pYDA18 | P*_PGK1_*_F | gcttttcttgaggatgggataagtgcatTTTGTTATATTTGTTGTAAAAAGTAGATAATTAC |
| pYDA19 | P*_PGK1_*_R | atgtagtgacaccgattatttaaagctgcaACGCACAGATATTATAACATCTG |
| pYDA20 | p416TEF1_F | GGCCGGTACCCAATTCG |
| pYDA21 | P*_TEF1_*_*YAS3*_t*_CYC1_*_F | ctcactatagggcgaattgggtaccggccGCAAATTAAAGCCTTCGAGC |
| pYDA22 | P*_TEF1_*_*YAS3*_t*_CYC1_*_R | ctcactaaagggaacaaaagctggagctcCAAAATGTTTCTACTC |
| pYDA23 | P*_PGK1_*_F2 | cctcactaaagggaacaaaagctggagctcGGAAGTACCTTCAAAGAATG |
| pYDA24 | P*_PGK1_*_R2 | TTGTTTTATATTTGTTGTAAAAAGTAG |
| pYDA25 | GFP_Fusion_F1 | atctactttttacaacaaatataaaacaaATGCGAATCCCCGGGTTAATTAAC |
| pYDA26 | GFP_Fusion_R1 | ggatctggagtccatagaaccaccaccTTTGTATAGTTCATCCATGCCATGTG |
| pYDA27 | *YAS1*_Fusion_F1 | CTATACAAAGGTGGTGGTTCTATGGACTCCAGATCCGCC |
| pYDA28 | *YAS1*_Fusion_R1 | gctttctcaggtatagcatgaggtcgctctTAGACTGGACTTTCTTCCTTCTTGACC |
| pYDA29 | t*_ADH1_*_F | GAGCGACCTCATGCTATACC |
| pYDA30 | t*_ADH1_*_R | actcactatagggcgaattgggtaccggccCGAATTTCTTATGATTTATGATTTTTATTATTAAATAAG |
| pYDA31 | GFP_Fusion_R2 | atgggataagtgcatagaaccaccaccTTTGTATAGTTCATCCATGCC |
| pYDA32 | *YAS2*_Fusion_F1 | ctatacaaaggtggtggttctATGCACTTATCCCATCCTC |
| pYDA33 | *YAS2*_Fusion_R1 | ctcactatagggcgaattgggtaccggcccTTGACGTGGCTGAACAAC |
| pYDA34 | *YAS3*_Fusion_R1 | ATGCCTAAGGCCTTGTC |
| pYDA35 | GFP_Fusion_F2 | gatgacaaggccttaggcatagaaccaccaccTTTGTATAGTTCATCCATGCC |
| pYDA36 | GFP_Fusion_R3 | ctaatctaagttttaattacaaaATGCGAATCCCCGG |
| pYDA37 | P*_TEF1_*_Fusion_F1 | cccggggattcgcatTTTGTAATTAAAACTTAGATTAGATTGCTATGC |
| pYDA38 | P*_TEF1_*_Fusion_R1 | ctcactaaagggaacaaaagctggagctccCAAAATGTTTCTACTCCTTTTTTAC |
| pYDA39 | *YAS1*_Fusion_F2 | atctactttttacaacaaatataaaacaaATGGACTCCAGATCCGCC |
| pYDA40 | *YAS1*_Fusion_R2 | cccggggattcgcatagaaccaccaccGACTGGACTTTCTTCCTTCTTGACC |
| pYDA41 | GFP_Fusion_F3 | gtccagtcggtggtggttctATGCGAATCCCCGGGTTAATTAAC |
| pYDA42 | GFP_Fusion_R3 | gctttctcaggtatagcatgaggtcgctcCTATTTGTATAGTTCATCCATGCCATG |
| pYDA43 | *YAS2*_Fusion_F2 | atctactttttacaacaaatataaaacaaATGCACTTATCCCATCCTC |
| pYDA44 | *YAS2*_Fusion_R2 | cccggggattcgcatagaaccaccaccTTCGTCGATCTTAGGAGG |
| pYDA45 | GFP_Fusion_F4 | gatcgacgaaggtggtggttctATGCGAATCCCCGGGTTAATTAAC |
| pYDA46 | GFP_Fusion_R4 | tacactactaatggcCTATTTGTATAGTTCATCCATGCCATG |
| pYDA47 | *YAS3*_Fusion_F | GGCGTCACCCATTTC |
| pYDA48 | GFP_Fusion_F5 | atgtaagcgtgacataactaattacatgaCTATTTGTATAGTTCATCCATGCCATG |
| pYDA49 | GFP_Fusion_R5 | ccgaccaagatgtcgaaatgggtgacgccggtggtggttctATGCGAATCCCCGGGTTAATTAAC |
| pYDA50 | *YAS1*_Fusion_R3 | cccggggattcgcatagaaccaccaccaacttttctctttttcttaggGACTGGACTTTCTTCCTTCTTG |
| pYDA51 | GFP_Fusion_F6 | gaaaagttggtggtggttctATGCGAATCCCCGGGTTAATTAAC |
| pYDA52 | GFP_Fusion_R6 | ccgaccaagatgtcgaaatgggtgacgcccctaagaaaaagagaaaagttggtggtggttctATGCGAATCCCCGGGTTAATTAAC |
| pYDA53 | t*_PDC6_*_F | gaactatacaaatagGCCATTAGTAGTGTACTCAAACG |
| PYDA54 | t*_PDC6_*_*YAS2*_F | tatacacatgtatatatatcgtatgctgcaTTGACGTGGCTGAACAAC |
| pYDA55 | P*_PGK1_*_F3 | tatacacatgtatatatatcgtatgctgcagACGCACAGATATTATAACATCTGC |
| pYDA56 | t*_ADH1_*_F2 | atgtagtgacaccgattatttaaagctgcaCATGCCGGTAGAGGTGTG |

^1^lower case letters are overlapping regions
