# Supplementary material for: Does co-expression of Yarrowia lipolytica genes encoding Yas1p, Yas2p and Yas3p make a potential alkane-responsive biosensor in Saccharomyces cerevisiae?
Source: PLoS One. 2020 Dec 17;15(12):e0239882. doi: 10.1371/journal.pone.0239882 (PMC7745969; doi:10.1371/journal.pone.0239882)
Supplement: S1 File — (DOCX) [file pone.0239882.s006.docx]

**Sequences**

Start codon, Stop codon, ARE1 (CTTGTGCGATGACAACAGCATGTG), Part of ARE1 found in P*_CYC1_* (CATGTG)

**Genscript_001: P*_PGK1_*-*YAS1* (codon optimized)-T*_ADH1_***

ACGCACAGATATTATAACATCTGCACAATAGGCATTTGCAAGAATTACTCGTGAGTAAGGAAAGAGTGAGGAACTATCGCATACCTGCATTTAAAGATGCCGATTTGGGCGCGAATCCTTTATTTTGGCTTCACCCTCATACTATTATCAGGGCCAGAAAAAGGAAGTGTTTCCCTCCTTCTTGAATTGATGTTACCCTCATAAAGCACGTGGCCTCTTATCGAGAAAGAAATTACCGTCGCTCGTGATTTGTTTGCAAAAAGAACAAAACTGAAAAAACCCAGACACGCTCGACTTCCTGTCTTCCTATTGATTGCAGCTTCCAATTTCGTCACACAACAAGGTCCTAGCGACGGCTCACAGGTTTTGTAACAAGCAATCGAAGGTTCTGGAATGGCGGGAAAGGGTTTAGTACCACATGCTATGATGCCCACTGTGATCTCCAGAGCAAAGTTCGTTCGATCGTACTGTTACTCTCTCTCTTTCAAACAGAATTGTCCGAATCGTGTGACAACAACAGCCTGTTCTCACACACTCTTTTCTTCTAACCAAGGGGGTGGTTTAGTTTAGTAGAACCTCGTGAAACTTACATTTACATATATATAAACTTGCATAAATTGGTCAATGCAAGAAATACATATTTGGTCTTTTCTAATTCGTAGTTTTTCAAGTTCTTAGATGCTTTCTTTTTCTCTTTTTTACAGATCATCAAGGAAGTAATTATCTACTTTTTACAACAAATATAACAAA**ATG**GACTCCAGATCCGCCAGTAACTCAGCCACACCACCTACAGCCGGTTCCCCTGAATCCGCCAAGAAGTCAGCCAGAAGAAAGTCGACTGTACAACCAATGCCAAGAGCACAAAAACCAAAGGGTGAATTGTTGACAGTTGAAGAAAAGAAAGCTAACCATATCGCATCTGAACAAAAGAGAAGACAAGCAATCAGAGAAGGTTTCGAAAGAATCACCAAGATCGTCCCTAACTTGGATAAGTCTCAAGGTAGATCAGAAGCCATAGTTTTAAATAAGACTGTCGCCTTCTTGAAGAACTTAATAGCTGAGGGTAAAGAATTGGAATTAAGATGTAATGAATTGCAAATTGTTACCCCACCACCAGCAGGTATGTCCAAGGAAAAGGTCAAGAAGGAAGAAAGTCCAGTC**TAA**GCGAATTTCTTATGATTTATGATTTTTATTATTAAATAAGTTATAAAAAAAATAAGTGTATACAAATTTTAAAGTGACTCTTAGGTTTTAAAACGAAAATTCTTATTCTTGAGTAACTCTTTCCTGTAGGTCAGGTTGCTTTCTCAGGTATAGCATGAGGTCGCTCTTATTGACCACACCTCTACCGGCATG

**Genscript_002: *YAS2* (codon optimized)-T*_PDC6_***

**ATG**CACTTATCCCATCCTCAAGAAAAGCAAGTCTTTATTACCACTCCATCCCCTAGTTCATCCGTCTCCACCCAATCCACTGCCTCCTCTTCATTGTCTTTATTTCATACAGCCGATTCAGACTTGTCAGCTTCCGAAGAAGATGACTTCCCATATCACGCTACTACACATACCAATATCAAACCTGGTGTAAGTTTTTCTGCAACTTCTGGTACAATGATGAGTGCCTTATCTCCAGTCTCAGTACAATCACCGTCTACCGTTTTCATGATGAAGGAACCAGCACAAGCCCAAGCTCAAATGTCTCATGATAATAACAACAACAACAACTCCAGTTCTCACGTTACCACTCCAAAACATAACGCTGCTCCTGTAACTAAGTCTGATGACCCATTTGATGTCGACAACTACCCTACCGATTTTGACTCTGCATTCTTGAATTTGTCACCAACTATGGGTATGGGTCCTGGTGACGGTATGGACGGTATTCCATTGTTCAATGCAGAAGAAGAATCAGCCTTTTCATCCTTCTTGGATAACGTTGCATTAGACCCAAACTTCATCTTCGAACCTAATTTGTCTGATGCCTTACCAAAATGGCCTGAATCCAAGCCTTGGGGTCCTAACGATAGTCATAAACCAAGAACCGACAAGTTTACTAAGTTCGGTAATTCTCCAGGTTTGGATTTGTTAGCCGCTAACTCACCTGACGCTGTTAACATACACGAAAGATTGTTGAAGCATGAATCTAAGGAATATTTGTCACCAAACCATAACGATTACGCACAAAGTGATTCAAGAGCACAAGCATTGGCTATGGCAGAAACACAAGGTTATGGTCAATTAAAACACAAGGAATCCAGAAGTTCTTTGAGTCAACCTTCATCCTTACATAATAACTCCGTTACCGGTAGTTCTGTCACAACCACTAGACCAAGTCCTCAAACTTCCGGTCCAAGTCCTGTTTCATCCGGTACATCTGCTGCTTCTTCTGCTCCAATGGGTTTACATTTTGGTTCAGATCCTGCATTTGGTGGTTCATCCTTCCAACCAAACTCCGGTACACCTGCCTTGAAAAAGGTCAGAGGTTTCGATGAATTACCACCTGCTGTACATGAAGGTCATGGTCACATGCACATGCAACCACAAAGAATGGTTACTGGTTTGGAAGGTGTTGTCAATCCTAAATTGGTAGTTGCTGCTGCTCAAATGTCTCAAGGTGTCACAGATCAAAACATTTTTATGTTGAAGAGAAGAAAGTCAGATAATATGGCTGCATCTATGAACTCTAACGTCCCACATGATATCTATGCTCCAGTACCTCACGCAGAACAAATGTACCATATGCAGCAGCAACAACAACAACAACACTTACATCAACAGCAGCAACAACAACACCACCAACAATCACAAAATCAACACATACAACAGCAACAGCAACAGCAACAACATCAAATGCATCACCCACATCACACCCAACAACACTTTCAAGCTAGAATGCATTTCGGTGACGGTATGGATGGTGAAGTATCAATGTCCACTGTTTCTCAAGCAGGTTTGCACATGAACTCTAACCCAAGTATGTTGTTTCCTGATAAAGACGCCTTAGCTGATTCATACCAACAACAACAACAACAAATGCACTCCCAACATAACCCACCTAGTCATCACCCACAACATAATCAACAGCAACAGCAACAACCGCAAGTCAAAACAGAACAAAATATGAGTGCTTCTCCAACACCTGGTTCACCAAACTTGACCGAAGATCAAAAGAGAATGAACCATATCAGTTCTGAAAAGAGAAGAAGAGATTTGATTAAACAAGAATTTGAAGAAATGTGTGGTTTGGTTCCAAGATTAGCCGCTAATTCTGATGAAAAGGGTAAAAGAAGACACGGTCATAGAGGTAGAATGCCTAAAGATTCTGACAAAGATAAGGACACAGGTACTAAATCAAAGTCCATATTGTTGTCTATCGTTTACGAATACATGTGCGAATTAGTCGAAAGAAATAAGGCTATGCGTGGTATGATTACTGAAAAGGGTGGTTACCATAGTGACATCGCCAACGCATTACATCCTCCTAAGATCGACGAA**TGA**GCCATTAGTAGTGTACTCAAACGAATTATTGTTGCAAATAAATAAACTTACACAGTTTGAATACATAAATCAATCAGACAAATAAATACATCGGTTCAAATTATACTAAATCTAAATACTACGTTATCGCCGTGAATTACGCAATTCGCATGTTACGTACTGCGCGTCTCTTGTTGAATATTTACCAATTGGGAAAAAGAACTCGTATTTCATTCCCCTTTTTGGAAAGGGGTGGGGAGAGACTGTTGTTCAGCCACGTCAA

**Genscript_003: P*_TEF1_*-*YAS3* (codon optimized)-T*_CYC1_***

CAAAATGTTTCTACTCCTTTTTTACTCTTCCAGATTTTCTCGGACTCCGCGCATCGCCGTACCACTTCAAAACACCCAAGCACAGCATACTAAATTTCCCCTCTTTCTTCCTCTAGGGTGTCGTTAATTACCCGTACTAAAGGTTTGGAAAAGAAAAAAGAGACCGCCTCGTTTCTTTTTCTTCGTCGAAAAAGGCAATAAAAATTTTTATCACGTTTCTTTTTCTTGAAAATTTTTTTTTTTGATTTTTTTCTCTTTCGATGACCTCCCATTGATATTTAAGTTAATAAACGGTCTTCAATTTCTCAAGTTTCAGTTTCATTTTTCTTGTTCTATTACAACTTTTTTTACTTCTTGCTCATTAGAAAGAAAGCATAGCAATCTAATCTAAGTTTTAATTACAAA**ATG**CCTAAGGCCTTGTCATCTAAGTCATTAGACGCCGCATCCATTCACATGGCATCCAACGGTGCCCCACCATTGATCCAAAAGTCATCCTTACACGACTTCGAAAGATTAAAATCAGGTATAGATGACATTCAAAATAGTGATACCAACGGTACACAATACTCCGTTGATGTCGGTTCCCAAGGTTTAAGAATGAGAATCCAAACCCAAGGTTACGCACCATCCGGTAATTCCAACAGAGGTTCACCAGTTCCTCCATCCCCTGCTTTATCAACGTCGACCTCTTCAGGTTCCACAAGTGCAGCTGCCTCAAGATCAACATCCTCACCTGTTCCACAACAACCTCAAACAGCTGCCACAGCAGCTGGTGGTCCAAGACCTGGTCAACCAAGATCAGCTTGGCAAGAAGTATTAATCTCAGCAACTAGTTTAGCCTCATTGAGTCAAGATTCCAGAAAAAGATTAAGATATTGTTTACATTTGTTGAAATTAGCAAACGCACACTTAGCTTCAACCGTTACTAAATTGCAAGGTGCAATCGCCGAAGAAACTGCTTATTCCTTGGCACAATCCATTGCCGCAAATCATGTCCCACACAATGAAAGACAAGCATATTTGCACCAACCTCCATCAGCAGAACCAGCCTTGTCCATCACAGCATTGAAGGCAGACGTAGTTTCAACAATTAGAAAAATCATCAAGGTTGTATCACAATACGCCGGTAATTCCTTGCCAGAACCTGCAAGATCCCATATCAGAACATACATATTGGGTTTACCATCCAGATGGGCTTCAACAACCGCATCAACTAATATTACACCAACCAGATCACCTGCTGGTTCACAATCCCCAGTTGGTTCCCCAAAAGAATGTGTCACACCTGACCATCAAGGTCCTCCTTTGCCAACAAACACAGTAAGTTCCCCAGTTCCTGAAGGTCCTTCAGATGAACAATTGTCCAGACACAGAATCGAAGTAGAAGCAGGTGGTAAAGTTTTGATCTTAGCAAACGAAGCATTGGACATGTTGGGTAACATTATCTCAATAGTTGATGGTACATTGGAAAGAGCAGAAGGTTGGTGTGAAGGTATCAACAGAGTTAAGCAAAGAGTTGGTTTGGGTGAAACAGCACCTGGTGCAGCTGGTGAACCAGGTGCTTCAACTGAAGCATCAGCTGAAGCATCAGCCGAAGCATCAGCAAGTGCCGCTGCTGCTTATTCTGCCGTTGATACCTCTACCGACCAAGATGTCGAAATGGGTGACGCCTGAATTAGTTATGTCACGCTTACATTCACGCCCTCCTCCCACATCCGCTCTAACCGAAAAGGAAGGAGTTAGACAACCTGAAGTCTAGGTCCCTATTTATTTTTTTTAATAGTTATGTTAGTATTAAGAACGTTATTTATATTTCAAATTTTTCTTTTTTTTCTGTACAAACGCGTGTACGCATGTAACATTATACTGAAAACCTTGCTTGAGAAGGTTTTGGGACGCTCGAAGGCTTTAATTTG

**P*_CYC1_***

AATTTTTTTGGAAAACCAAGAAATGAATTATATTTCCGTGTGAGACGACATCGTCGAATATGATTCAGGGTAACAGTATTGATGTAATCAATTTCCTACCTGAATCTAAAATTCCCGGGAGCAAGATCAAGATGTTTTCACCGATCTTTCCGGTCTCTTTGGCCGGGGTTTACGGACGATGGCAGAAGACCAAAGCGCCAGTTCATTTGGCGAGCGTTGGTTGGTGGATCAAGCCCACGCGTAGGCAATCCTCGAGCAGATCCGCCAGGCGTGTATATATAGCGTGGATGGCCAGGCAACTTTAGTGCTGACACATACAGGCATATATATATGTGTGCGACAACACATGATCATATGGCATGCATGTGCTCTGTATGTATATAAAACTCTTGTTTTCTTCTTTTCTCTAAATATTCTTTCCTTATACATTAGGACCTTTGCAGCATAAATTACTATACTTCTATAGACACACAAACACAAATACACACACTAAATTAATA

**P*_CYC1_* with ARE1 (Position 1)**

AATTTTTTTGGAAAACCAAGAAATGAATTATATTTCCGTGTGAGACGACATCGTCGAATATGATTCAGGGTAACAGTATTGATGTAATCAATTTCCTACCTGAATCTAAAATTCCCGGGAGCAAGATCAAGATGTTTTCACCGATCTTTCCGGTCTCTTTGGCCGGGGTTTACGGACGATGGCAGAAGACCAAAGCGCCAGTTCATTTGGCGAGCGTTGGTTGGTGGATCAAGCCCACGCGTAGGCAATCCTCGAGCAGATCCGCCAGGCGTGTATATATAGCGTGGATGGCCAGGCAACTTTAGTGCTGACACATACTTGTGCGATGACAACAGCATGTGCAGGCATATATATATGTGTGCGACAACACATGATCATATGGCATGCATGTGCTCTGTATGTATATAAAACTCTTGTTTTCTTCTTTTCTCTAAATATTCTTTCCTTATACATTAGGACCTTTGCAGCATAAATTACTATACTTCTATAGACACACAAACACAAATACACACACTAAATTAATA

**P*_CYC1_* with ARE1 (Position 2)**

AATTTTTTTGGAAAACCAAGAAATGAATTATATTTCCGTGTGAGACGACATCGTCGAATATGATTCAGGGTAACAGTATTGATGTAATCAATTTCCTACCTGAATCTAAAATTCCCGGGAGCAAGATCAAGATGTTTTCACCGATCTTTCCGGTCTCTTTGGCCGGGGTTTACGGACGATGGCAGAAGACCAAAGCGCCAGTTCATTTGGCGAGCGTTGGTTGGTGGATCAAGCCCACGCGTAGGCAATCCTCGAGCAGATCCGCCAGGCGTGTATATATAGCGTGGATGGCCAGGCAACTTTAGTGCTGACACATACAGGCATATATATATGTGTGCGACAACACATGATCATATGGCATGCATGTGCTCTCTTGTGCGATGACAACAGCATGTGGTATGTATATAAAACTCTTGTTTTCTTCTTTTCTCTAAATATTCTTTCCTTATACATTAGGACCTTTGCAGCATAAATTACTATACTTCTATAGACACACAAACACAAATACACACACTAAATTAATA

**P*_CYC1_* with ARE1 (Position 1,2)**

AATTTTTTTGGAAAACCAAGAAATGAATTATATTTCCGTGTGAGACGACATCGTCGAATATGATTCAGGGTAACAGTATTGATGTAATCAATTTCCTACCTGAATCTAAAATTCCCGGGAGCAAGATCAAGATGTTTTCACCGATCTTTCCGGTCTCTTTGGCCGGGGTTTACGGACGATGGCAGAAGACCAAAGCGCCAGTTCATTTGGCGAGCGTTGGTTGGTGGATCAAGCCCACGCGTAGGCAATCCTCGAGCAGATCCGCCAGGCGTGTATATATAGCGTGGATGGCCAGGCAACTTTAGTGCTGACACATACTTGTGCGATGACAACAGCATGTGCAGGCATATATATATGTGTGCGACGACACATGATCATATGGCATGCATGTGCTCTCTTGTGCGATGACAACAGCATGTGGTATGTATATAAAACTCTTGTTTTCTTCTTTTCTCTAAATATTCTTTCCTTATACATTAGGACCTTTGCAGCATAAATTACTATACTTCTATAGACACACAAACACAAATACACACACTAAATTAATA

**P*_ALK1_* (Amplified from *Yarrowia lipolytica*)**

CAGTGATGAGGACACACTCTAGGACGTCTGGTACCACAAGGAGGGGCCAACTGTCGCTGTCATCGCTGTCTCCTGGACAGCAGAGCTAACTGTTGTACTCCAGTGACCAACCAAAATTCTTCTAATGTTGCGGCTCAAGGTCTGTCCCCACAACTGTTGAAAGCCTAAGCGTCATGGTAACAACGAGGAACAAGGGCTTTTCGAACCTTGTGCGATGACAACAGCATGTGAATAAGTGTTAGTGGGGAAGATTCAAGACAGCAGAAAGTTAGCGGGTGTAAGGGGGGGAGGACCAGAGGGGGTGTTAACTCATCAGAACCTTTCCTGCCGAGATGTCAGCAATCAATTCGCCTTCCATACATCTTATGATGCTATAGATTCCAGTTCTGAGGTGTTCCTGGTATGTTTTCATCTTCTTTCATTCCATTCGAGATCCCTCAAGAGTGCATGTAAACTGAAACCTTATGCCAAACTGAGCGATCGTGAATATGAAAAAGTCTGGGAAAGCGTCAATTCAAAAAAGCGAACAAAAAAAGCACAGAGGTATATATATAGGTGACAGCACCAAACCATAGGTCCTCCCCAGAATACTCCTGCACT
